# Itraconazole-Induced Increases in Gilteritinib Exposure Are Mediated by CYP3A and OATP1B

**DOI:** 10.3390/molecules27206815

**Published:** 2022-10-12

**Authors:** Dominique A. Garrison, Yan Jin, Zahra Talebi, Shuiying Hu, Alex Sparreboom, Sharyn D. Baker, Eric D. Eisenmann

**Affiliations:** 1 Division of Pharmaceutics and Pharmacology, The Ohio State University, Columbus, OH 43210, USA; 2 Division of Outcomes and Translational Sciences, The Ohio State University, Columbus, OH 43210, USA

**Keywords:** gilteritinib, OATP1B, itraconazole, pharmacokinetics, drug–drug interactions, CYP3A

## Abstract

Gilteritinib, an FDA-approved tyrosine kinase inhibitor approved for the treatment of relapsed/refractory FLT3-mutated acute myeloid leukemia, is primarily eliminated via CYP3A4-mediated metabolism, a pathway that is sensitive to the co-administration of known CYP3A4 inhibitors, such as itraconazole. However, the precise mechanism by which itraconazole and other CYP3A-modulating drugs affect the absorption and disposition of gilteritinib remains unclear. In the present investigation, we demonstrate that pretreatment with itraconazole is associated with a significant increase in the systemic exposure to gilteritinib in mice, recapitulating the observed clinical drug–drug interaction. However, the plasma levels of gilteritinib were only modestly increased in CYP3A-deficient mice and not further influenced by itraconazole. Ensuing in vitro and in vivo studies revealed that gilteritinib is a transported substrate of OATP1B-type transporters, that gilteritinib exposure is increased in mice with OATP1B2 deficiency, and that the ability of itraconazole to inhibit OATP1B-type transport in vivo is contingent on its metabolism by CYP3A isoforms. These findings provide new insight into the pharmacokinetic properties of gilteritinib and into the molecular mechanisms underlying drug–drug interactions with itraconazole.

## 1. Introduction

Gilteritinib is the first and only FDA-approved fms-like tyrosine kinase 3 (FLT3) inhibitor for the treatment of adults with relapsed or refractory acute myeloid leukemia (R/R AML) with a FLT3 mutation as a monotherapy [1,2,3]. In the Phase III ADMIRAL trial, adult patients with R/R FLT3-mutated AML treated with gilteritinib had a significantly longer median overall survival and higher response rates compared with patients receiving standard salvage chemotherapy [4,5]. Based on these positive outcomes, gilteritinib is undergoing further investigation in other clinical settings in combination with chemotherapy or other agents for treatment and maintenance of FLT-mutated AML [6,7]. However, despite its clinical efficacy, the use of gilteritinib is negatively influenced by extensive inter-individual pharmacokinetic variability and by adverse events that may lead to dose reductions or discontinuation of treatment [4]. Elucidating the mechanisms that govern the pharmacokinetic properties of gilteritinib could potentially improve dosing strategies, minimize adverse reactions, and prevent the occurrence of potentially harmful drug–drug interactions (DDIs).

Previous in vitro studies have indicated that gilteritinib is primarily eliminated via CYP3A-mediated metabolism [8,9]. Furthermore, a Phase I open-label parallel-group study demonstrated that the concomitant use of strong and moderate CYP3A inhibitors or strong CYP3A inducers alters measures of systemic exposure to gilteritinib [10]. However, doubts about the causal relation of CYP3A4-mediated metabolism with these pharmacokinetic interactions have been raised due to the recognition that the perpetrator drugs often affect other elimination pathways, including xenobiotic OATP1B-type transporters [11], that may be relevant to the hepatic uptake of gilteritinib [12]. The aim of this study was to characterize the relative contribution of CYP3A-mediated metabolism and OATP1B-mediated transport to the pharmacokinetics and DDI liabilities of gilteritinib.

## 2. Results

### 2.1. Contribution of CYP3A to the Interaction between Gilteritinib and Itraconazole

**Figure 1:**
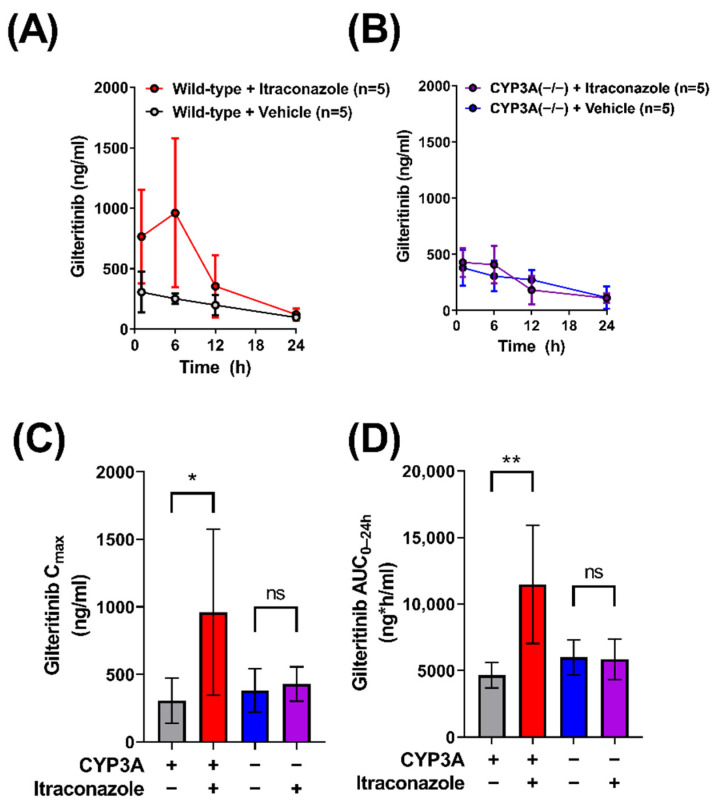
Itraconazole-related increases in gilteritinib exposure are dependent on CYP3A function, but not necessarily CYP3A inhibition. Plasma concentrations of gilteritinib in (**A**) wild-type mice and (**B**) CYP3A(−/−) mice with or without itraconazole (100 mg/kg) given 30 min before gilteritinib (10 mg/kg). Samples were analyzed via LC-MS/MS. The maximum observed concentration (C_max_) (**C**) and area under the concentration–time curve (AUC) (**D**) from time zero to the last collected and 24 h (AUC_0–24h_) were calculated with non-compartmental analysis using Phoenix WinNonlin 8.1. Data are shown as mean (bars) and SD (error bars). * *p* < 0.05, ** *p* < 0.01. ‘ns’ denotes not statistically significant.

Clinical data from a Phase 1 open label study indicated that gilteritinib exposure is increased 2.2-fold when co-administered with itraconazole, a known CYP3A4 inhibitor, in humans [10]. To demonstrate that this DDI can be recapitulated in mice, we evaluated the pharmacokinetic profile of gilteritinib in male wild-type mice given gilteritinib (10 mg/kg) alone or in combination with itraconazole (100 mg/kg). As expected, elevated levels of gilteritinib were observed in plasma of wild-type mice given itraconazole with gilteritinib (Figure 1A), and the AUC_0–24h_ was increased by about 2.5-fold (*p* < 0.05; Table 1), consistent with the observed clinical pharmacokinetic data demonstrating a 2.2-fold increase in gilteritinib exposure when given with itraconazole [10].

Previous in vitro studies have indicated that CYP3A4 is the major enzyme involved in the metabolism of gilteritinib [8]. To investigate the in vivo contribution of CYP3A isoforms to the metabolism of gilteritinib, we conducted comparative pharmacokinetic studies in wild-type mice and CYP3A(−/−) mice receiving oral gilteritinib (10 mg/kg) (Figure 1B, Table 1). Unexpectedly, similar C_max_ values were observed in wild-type mice and CYP3A(−/−) (Figure 1C), accompanied by a minor 1.3-fold difference in AUC_0–24h_ (Figure 1D). To investigate whether gilteritinib exposure would increase by treatment with itraconazole in the absence of CYP3A, we administered gilteritinib (10 mg/kg) with itraconazole (100 mg/kg) to CYP3A(−/−) mice (Figure 1B). No differences were observed in either C_max_ or AUC_0–24h_ values (Figure 1C,D). Importantly, regardless of treatment with itraconazole, CYP3A(−/−) mice had exposure parameters comparable to wild-type mice that did not receive itraconazole. Based on previous investigations demonstrating that, unlike the parent compound, itraconazole metabolites formed via CYP3A-mediated biotransformation have inhibitory properties toward OATP1B-type transporters [13], we next hypothesized that OATP1B-type transport contributes to the DDI between itraconazole and gilteritinib.

### 2.2. Interaction of Gilteritinib with OATP1B-Type Transporters

**Figure 2:**
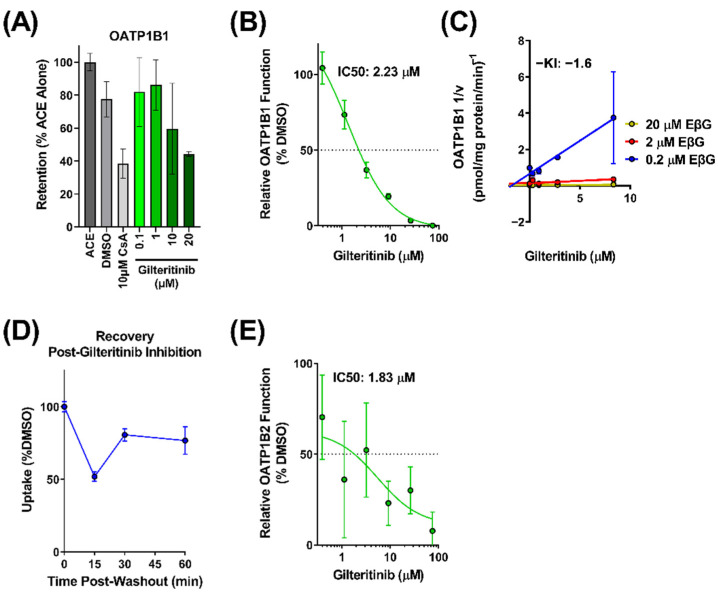
Gilteritinib is a substrate and inhibitor of OATP1B-type transporters. (**A**) Uptake of gilteritinib by OATP1B1 overexpressing HEK293 cells was assessed via competitive counterflow assay. Uptake was measured by accumulation of the fluorescent substrate 8-acetoxypyrene-1,3,6-trisulfonate (ACE) with or without gilteritinib. (**B**) Inhibitory potency of gilteritinib on OATP1B1 overexpressing HEK293 cells compared to vector control cells. Inhibition was determined by the intracellular accumulation of the radiolabeled probe substrate E*β*G in the presence of gilteritinib at increasing concentrations (0.5–75 µM). (**C**) Dixon plot showing various concentrations of radiolabeled E*β*G (0.2, 2, 20 µM) uptake in the presence of gilteritinib (0.3–8.3 µM) in OATP1B1 overexpressing HEK293 cells (**D**) Recovery of OATP1B1 function after 15 min treatment of gilteritinib (15 µM). (**E**) Inhibitory potency of gilteritinib on OATP1B2 overexpressing HEK293 cells compared to vector control cells. Inhibition was determined by the intracellular accumulation of 8-FcA. Data are shown as mean (bars or symbols) and SD (error bars).

Since little is known about the interaction of gilteritinib with OATP1B-type transporters, we provided a more comprehensive evaluation of potential interactions. We first examined the in vitro transporter-mediated cellular uptake of gilteritinib. Based on known difficulties associated with direct measurement of the transport of targeted anticancer agents [13], we performed a competitive counterflow assay to identify gilteritinib as a transported substrate of OATP1B1 (Figure 2A). Using this method, OATP1B1 overexpressing cells and vector control cells were exposed to various concentrations of gilteritinib (0.1–20 μM). The competitive counterflow assay measures the uptake of a test compound by measuring the concentration of the fluorescent indicator substrate 8-acetoxypyrene-1,3,6-trisulfonate (ACE) that is displaced through an exchange mechanism via the OATP1B1 transporter [14,15]. Therefore, gilteritinib uptake was determined indirectly by the efflux of ACE out of the cells, with data normalized to ACE alone. Cyclosporine A, a known OATP1B1 substrate, was used as a positive control and DMSO was used as a negative control. We found that gilteritinib decreased the fluorescent signal in cells in a concentration-dependent manner (Figure 2A), supporting the thesis that gilteritinib is a transported substrate of OATP1B1. 

To further characterize the interaction between gilteritinib and OATP1B1, we performed a radioactive uptake assay to measure the inhibitory potential of gilteritinib on OATP1B1 function. In overexpressing OATP1B1 cells and vector control cells, the cellular accumulation of the radiolabeled probe substrate E*β*G was measured in the presence of increasing concentrations of gilteritinib (0.4–75 μM). Gilteritinib inhibited the cellular accumulation of E*β*G in a concentration-dependent manner with an IC50 value of 2.23 µM. (Figure 2B). When the C_max_ at steady-state in patients (0.68 µM [10]) is divided by the IC50 (2.23 µM), the result is 0.3, a value that is considered clinically relevant [16]. To define the mechanism of inhibition and to determine the inhibition constant (Ki value) of gilteritinib on the function of OATP1B1, a Dixon plot was generated [17] in which the reciprocal velocity is plotted against the concentration of gilteritinib (0.3–8.3 µM) at three concentrations of the substrate E*β*G (0.2, 2, 20 µM). This analysis showed that gilteritinib inhibits E*β*G uptake by OATP1B1 with an estimated Ki of 1.6 µM (Figure 2C). Surprisingly, these data suggest that gilteritinib noncompetitively inhibits OATP1B transport of E*β*G. Nonetheless, this observation is consistent with other OATP1B substrates [18], and is potentially due to the presence of multiple binding sites on OATP1B1 [19]. The potent inhibition of OATP1B1 by gilteritinib may be related to inhibition of the protein kinase LYN [20,21,22], which mediates phosphorylation-mediated activation of OATP1B1 and OATP1B3. Washout experiments demonstrated that the inhibitory mechanism observed for gilteritinib was reversible, with 75% of transport recovered within a 1-h period (Figure 2D).

The interaction of gilteritinib with OATP1B-type transporters was next evaluated for OATP1B2, the mouse orthologue for human OATP1B1 and OATP1B3, using 8-FcA as a fluorescent probe substrate [23]. Using OATP1B2-overexpressing cells and vector control cells, we found that gilteritinib inhibited OATP1B2 function with an IC50 value of 1.83 µM (Figure 2E). Altogether, these in vitro data strongly suggest that gilteritinib is both a substrate and inhibitor of OATP1B-type transporters.

### 2.3. Contribution of OATP1B to the Interaction between Gilteritinib and Itraconazole

**Figure 3:**
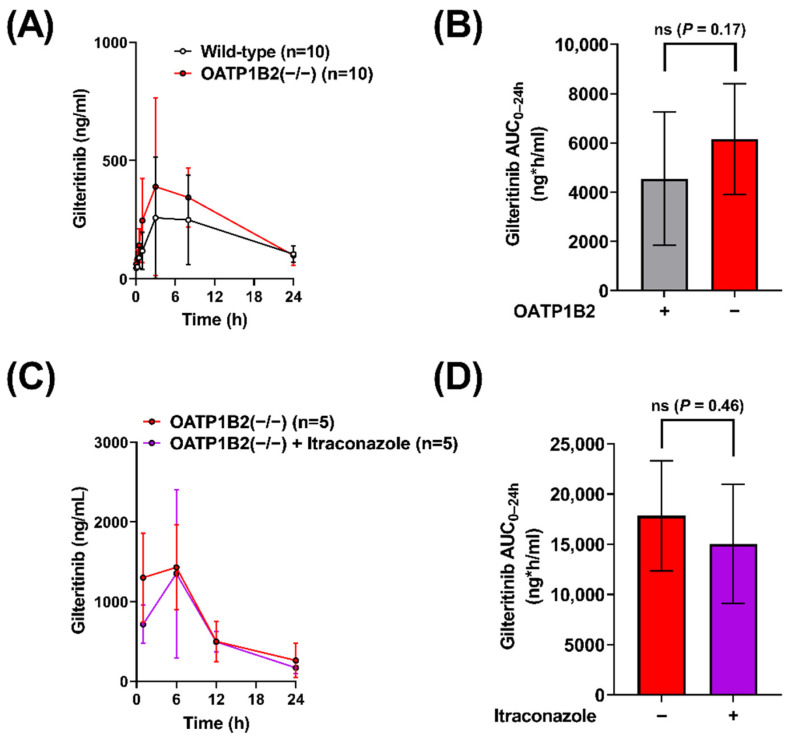
Itraconazole-related increases in gilteritinib exposure are dependent on OATP1B2. (**A**) Plasma concentrations of gilteritinib in wild-type mice and OATP1B2(−/−) mice administered gilteritinib (10 mg/kg). (**C**) Plasma concentrations of gilteritinib in OATP1B2(−/−) mice administered gilteritinib (10 mg/kg) alone or thirty minutes after itraconazole (100 mg/kg). Samples were analyzed via LC-MS/MS, and (**B**,**D**) the area under the concentration–time curve (AUC) using the last observed timepoint (AUC_0–24h_) was calculated with non-compartmental analysis using Phoenix WinNonlin 8.1. Data are shown as mean (bars or symbols) and SD (error bars). ‘ns’ denotes not statistically significant.

To evaluate the OATP1B-type transport of gilteritinib in vivo, we administered gilteritinib (10 mg/kg) to wild-type mice and OATP1B2(−/−) mice (Figure 3A) and found that OATP1B2-deficiency was associated with a 1.4-fold increase in gilteritinib AUC_0–24h_ (Table 1, Figure 3B). To provide a mechanistic explanation for this observation, we re-examined the inhibitory potential of itraconazole and its metabolites keto-itraconazole and hydroxy-itraconazole, on OATP1B1-mediated transport, by assessing E*β*G uptake in OATP1B1overexpressing cells and vector control cells. We found that keto-itraconazole and hydroxy-itraconazole inhibited OATP1B1-mediated transport of E*β*G, with IC50 values of 7.50 µM and 2.95 µM, respectively (Figure S1), whereas the parent compound itraconazole was less potent with an IC50 value of 27.3 µM. These data are in line with previous observations [11] and support the possibility that CYP3A-mediated metabolism of itraconazole is required for in vivo inhibition of OATP1B1.

To evaluate the OATP1B-mediated effects of itraconazole on the systemic exposure of gilteritinib in vivo, gilteritinib (10 mg/kg) was administered alone or in combination with itraconazole (100 mg/kg) to OATP1B2(−/−) mice (Figure 3C). There was no difference in gilteritinib exposure in OATP1B2(−/−) when administered alone or in combination with itraconazole (Table 1, Figure 3D), implying that the DDI between itraconazole and gilteritinib in wild-type mice is dependent on OATP1B2, despite the observed variability between experiments. This degree of unexplained experimental variability has been documented previously with other cancer drugs, including tyrosine kinase inhibitors [24], in both wild-type and OATP1B2(−/−) mice [25,26]. To verify that the observations in OATP1B2(−/−) mice are not due to compensatory deregulation of relevant ADME genes, we confirmed that the expression of the main CYP3A isoform, CYP3A11, in the livers of OATP1B2(−/−) mice and matched wild-type mice is unchanged (Figure S2A), as reported previously [13]. Similarly, expression of the OATP1B2 gene was similar in the livers of CYP3A(−/−) mice and wild-type mice (Figure S2B).

### 2.4. Impact of Itraconazole on Endogenous Biomarker of OATP1B Function

**Figure 4:**
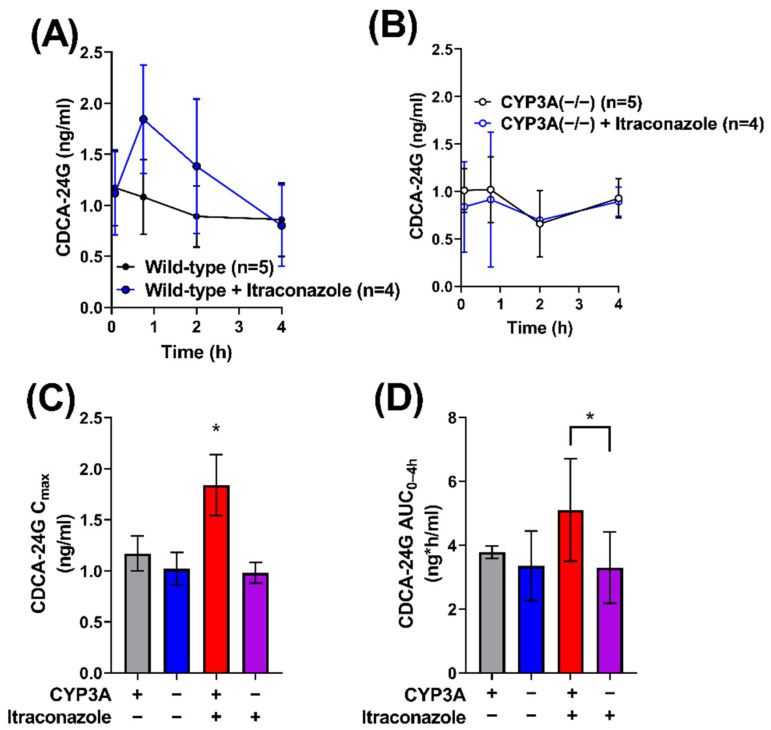
Itraconazole-induced increase in OATP1B biomarker is dependent on CYP3A. CDCA-24G concentrations were measured in serial plasma samples were collected and from female (**A**) wild-type and (**B**) CYP3A(−/−) mice that were untreated or treated with itraconazole. Samples were analyzed for concentrations of CDCA-24G, an endogenous biomarker of OATP1B transporter function, via LC-MS/MS, and the (**C**) maximum plasma concentration (C_max_) and (**D**) area under the concentration–time curve (AUC) using the last observed timepoint (AUC_0–4h_) were estimated with non-compartmental analysis (NCA) using Phoenix WinNonlin 8.1. Data are shown as mean (symbols or bars) and SD (error bars). ∗ *p* < 0.05.

Previous studies have revealed that the bile acid metabolite chenodeoxycholate 24-glucuronide (CDCA-24G) is an endogenous substrate of OATP1B-type transporters that is sensitive to treatment with the OATP1B inhibitor, rifampin [27,28]. To demonstrate that the impact of itraconazole on gilteritinib pharmacokinetics is dependent on CYP3A-mediated biotransformation of itraconazole to form metabolites that inhibit OATP1B1, the influence of itraconazole (100 mg/kg) on circulating levels of CDCA-24G was evaluated in wild-type mice and CYP3A(−/−) mice (Table 2). While there was no statistically significant difference in CDCA-24G levels between untreated and itraconazole-treated CYP3A(−/−) mice (Figure 4A–D), the CDCA24-G C_max_ (*p* < 0.05; Figure 4C) and AUC_0–24h_ (*p* < 0.05; Figure 4D) were elevated in wild-type mice treated with itraconazole when compared to untreated wild-type mice or CYP3A(−/−) mice treated with itraconazole. These results support our hypothesis that itraconazole-induced increases in gilteritinib exposure are dependent on OATP1B-type inhibition by itraconazole metabolites formed by CYP3A-mediated biotransformation. 

## 3. Discussion

In the present study, we further characterized the pharmacokinetic properties of gilteritinib, an FDA-approved tyrosine kinase inhibitor for the treatment of R/R FLT3-mutated AML. In particular, we investigated the contribution of CYP3A to the elimination of gilteritinib in mice and observed that the genetic deficiency of CYP3A isoforms does not substantially affect the pharmacokinetic handling of gilteritinib. In addition, we identified OATP1B-type transport as a contributor to the disposition of gilteritinib that is sensitive to genetic deficiency and pharmacological inhibition of OATP1B-type transporters by metabolites of itraconazole, an agent frequently selected as a preferred CYP3A4 inhibitor in clinical DDI studies. These studies shed light on the mechanisms involved in the pharmacokinetic properties of gilteritinib and have potential implications for the design of future DDI studies involving victim drugs that are dual substrates of CYP3A-mediated metabolism and OATP1B-mediated transport. 

Clinical studies have shown that increases in exposure to gilteritinib are associated with an increased incidence of adverse events and toxicity that often require dose reduction or interruption, which, in turn, may compromise the outcome of treatment. Indeed, treatment with gilteritinib is associated with potentially severe adverse events including arthralgia, dyspnea, and febrile neutropenia. The maximum tolerated dose of gilteritinib has been reported to be 200 mg [29] or 300 mg [4]. Given that gilteritinib has dose proportional pharmacokinetics [29] and is approved at a dose of 120 mg, a >two-fold increase in exposure is clinically relevant. Therefore, detailed understanding of the pharmacokinetic properties of gilteritinib and its potential liability to DDIs is of practical and clinical significance, especially in view of the numerous clinical trials that are being considered or already underway in which gilteritinib is given in combination with other agents. These studies include combinations of gilteritinib with standard chemotherapy (NCT05199051), azacitidine (NCT02752035), lanraplenib, an inhibitor of spleen tyrosine kinase (SYK) with potential immunomodulating and antineoplastic activities (NCT05028751), or venetoclax (NCT04140487). Indeed, combination treatment with venetoclax and gilteritinib is highly promising in relapsed/refractory AML [30] and is being investigated in newly diagnosed AML (NCT05520567). In this context, it is noteworthy that we previously also identified venetoclax as a potential OATP1B substrate [13], suggesting that there is a potential DDI between gilteritinib and venetoclax. Despite these ongoing combinatorial trials, knowledge on the sensitivity of gilteritinib to DDIs has been relatively limited, and prior studies have primarily focused on the inhibition of CYP3A-mediated metabolism in both human patients [10] and rats [31], as well as on interference with transport by the organic cation transporters MATE1 [10], OCT1, and OCT2 [32]. 

While in vitro studies have shown that CYP3A4 is the major metabolizing enzyme of gilteritinib, regulatory documents indicate that none of the three primary metabolites of gilteritinib exceeds 10% of total drug exposure and that the majority of gilteritinib is eliminated from the body unchanged. This suggests that the importance of CYP3A4-mediated metabolism to gilteritinib elimination may be less substantial than could be inferred from DDI studies indicating that itraconazole increases exposure to gilteritinib by 2.2-fold in humans [10] and 1.4-fold in rats [31]. The notion that a similar DDI was observed here in mice, combined with the observation that exposure to gilteritinib was unchanged in CYP3A(−/−) mice, suggests that alternative mechanisms contribute to the observed DDI. Indeed, a limitation to studying potential liability of DDIs is that many perpetrator drugs interact with multiple enzymes and transporters of putative relevance to the victim drug. This multiplicity complicates the ability to determine the mechanism by which an observed DDI occurs in vivo. If a perpetrator drug and a victim drug both have overlapping enzyme-transporter interplay in contributing to hepatic clearance, difficulties arise in extrapolating in vitro results to unstudied in vivo scenarios. This interplay emphasizes the need for extensive pharmacokinetic profiling of drugs both in vitro and in vivo and a need to carefully select agents for use in mechanistic DDI investigations [16,33].

Itraconazole has been extensively explored for its CYP3A4 inhibitory properties and is frequently used as a standard CYP3A inhibitor in DDI-directed studies. Although itraconazole is a known inhibitor of ABCB1 (P-glycoprotein)-mediated efflux [34,35,36], there is a relative paucity of data pertaining to its interaction with other xenobiotic transporters. Previous studies have shown that itraconazole metabolites, keto-itraconazole and hydroxy-itraconazole, potently inhibit OATP1B-type transport [11,13], and this prior knowledge is consistent with our present in vitro studies. Although the itraconazole metabolites are also more potent inhibitors of CYP3A function than the parent drug [35,37], this observation is of unlikely pertinence to the itraconazole-gilteritinib DDI given that even complete absence of CYP3A in mice was not associated with substantially altered elimination of gilteritinib. Regardless of the underlying mechanisms, the present study raises further concerns regarding the selectivity and specificity of enzyme inhibitors that are typically selected for clinical DDI studies when elimination pathways of the victim drug have not been adequately elucidated. 

To determine the rate-limiting step in the elimination of gilteritinib, we initially administered gilteritinib alone or in combination with itraconazole in OATP1B2-deficient mice and observed the lack of a DDI expected on the basis of results obtained in wild-type mice. This surprising finding supports the hypothesis that keto-itraconazole and hydroxy-itraconazole are responsible for in vivo inhibition of OATP1B1 function and the observed pharmacokinetic changes with gilteritinib when given in combination with itraconazole. This is consistent with the notion that itraconazole does not influence the systemic exposure to gilteritinib in a context of genetic deficiency of all CYP3A isoforms. Further investigation employing pre-treatment with keto-itraconazole and hydroxy-itraconazole would be required to definitively verify a causal connection of these metabolites with inhibition of OATP1-mediated transport of gilteritinib. Indeed, it remains possible that these metabolites act through additional mechanisms, such as increasing the intestinal uptake of gilteritinib (e.g., through inhibition of gilteritinib efflux). However, while it is clear that itraconazole impacts gilteritinib exposure, the employed sampling scheme limits our ability to differentiate between potential effects of itraconazole on gilteritinib absorption, distribution, and/or elimination.

The thesis that, in the presence of a dual CYP3A and OATP1B antagonist, systemic exposure to a substrate can be affected to a greater extent by OATP1B1 inhibition than by CYP3A4 inhibition is not unprecedented. For example, the elimination mechanisms of the HMG-CoA reductase inhibitor atorvastatin are dependent on hepatic uptake by OATP1B1 and subsequent metabolism by CYP3A, and sensitivity analyses have indicated that the uptake transport is a rate-limiting event that is much more sensitive (by ~6-fold) to inhibition than the metabolic pathway [38]. It should be pointed out that at least some previous studies indicated that the administration of itraconazole (100 mg) to human subjects was not associated with changes in the circulating levels of the OATP1B biomarkers coproporphyrin (CP) I and CPIII [39,40]. In addition, in a study exploring the relative importance of OATPs and CYP3A4 in the hepatic elimination of atorvastatin in vivo using a cassette microdose study, investigators have found that the dose-normalized AUC of atorvastatin increased 12-fold when co-administered with a single dose of rifampicin (an OATP1B inhibitor) but did not change by pre-treatment with itraconazole [41]. Although further studies are required to unravel the basis of these apparent paradoxes and its potential species- and substrate-dependence, it is worth pointing out that coproporphyrins [42,43] and atorvastatin [44] are substrates of ABCC2 (MRP2), a canalicular efflux transporter that is sensitive to inhibition by rifampin [45], and this recognition may compromise the ability to ascribe phenotypic observations to specific causal mechanisms. 

In the current study, we found that itraconazole-induced increases in gilteritinib exposure are partially dependent on CYP3A function, but not necessarily CYP3A inhibition, and that OATP1B-type transporters play a role in this DDI. Our findings indicate that the mechanisms underlying the ability of itraconazole to act as a perpetrator in DDIs are multifaceted and may involve both drug metabolizing enzymes and drug transporters. Moreover, our findings suggest hepatic OATP1B1-mediated transport should be taken into consideration in the evaluation of DDIs of gilteritinib with other agents, such as venetoclax, a combination that is currently under further investigation. These data provide further insight into the pharmacokinetic profile of gilteritinib and into the molecular mechanisms underlying DDIs with itraconazole.

## 4. Materials and Methods

### 4.1. Chemicals and Reagents

Gilteritinib was purchased from Sellekchem (Houston, TX, USA). Reference standards of gilteritinib and [^2^H_5_]-gilteritinib (gilteritinib-d5), used as an internal standard for the analytical method, were purchased from AlsaChim (Illkirch-Graffenstaden, France). Reagents for LC-MS/MS were as previously described [32]. Itraconazole was purchased from MedChemExpress (Monmouth Junction, NJ, USA). The itraconazole metabolites hydroxy-itraconazole and keto-itraconazole were kindly provided by Dr. Nina Isoherranen (University of Washington, Seattle, WA, USA). The [^3^H]-estradiol-17β-D-glucuronide (E*β*G; specific activity, 50.1 Ci/mmol) was purchased from American Radiolabeled Chemicals (Saint Louis, MO, USA). Standard cell-culturing procedures were conducted using Dulbecco’s Modified Eagle Media (DMEM) and fetal bovine serum (FBS) obtained from Gibco (Grand Island, NY, USA). Poly-D-lysine for coating plates was purchased from MP Biomedicals (Solon, OH, USA). Pierce BCA protein assay kits were purchased from Thermo Fisher Scientific, and 8-acetoxypyrene-1,3,6-trisulfonate (ACE) was obtained from Carbosynth Limited (Compton, Berkshire, UK).

### 4.2. Transport Inhibition Assays

Human embryonic kidney (HEK293) cells stably transfected with human OATP1B1 (SLCO1B1) cDNA, or an empty control vector were established and cultured as previously described [46]. Cells were maintained in DMEM supplemented with 10% FBS, 50 µg/mL hygromycin B, 15 µg/mL blasticidin, and maintained in a humidified incubator at 37 °C with 5% CO_2_. Prior to plating, 24-well tissue culture plates (Thermo Fisher Scientific) were coated with poly-d-lysine. Seeding media was prepared using phenol red-free DMEM with 10% FBS with no selection agents. To the seeding media, 1 µg/µL of doxycycline was added to induce expression of OATP1B1.

OATP1B1 overexpressing cells and vector control cells were plated in 24-well tissue culture plates at a volume of 0.5 mL (containing 2 × 10^6^ cells/mL) per well. Plates were returned to an incubator at 37 °C and transport assays were conducted 24 h later. Transport activity was measured using E*β*G, a prototypical substrate of OATP1B1. Experiments were conducted using phenol red- and serum-free DMEM. Intracellular levels of total radioactivity originating from E*β*G were assessed by liquid scintillation counting. The resulting counts were normalized to total protein levels as determined by a Pierce protein assay. The influence of gilteritinib, itraconazole, keto-itraconazole, or hydroxy-itraconazole on OATP1B1-mediated transport was assessed following a 15 min pre-incubation at various concentrations of test inhibitors followed by co-incubation of test inhibitors with E*β*G. Inhibition of transport activity by gilteritinib was determined by comparing the accumulation of E*β*G in the presence of gilteritinib, itraconazole, keto-itraconazole, or hydroxy-itraconazole to that of E*β*G in the presence of only the control vehicle, DMSO. The final concentration of DMSO in media was less than 0.2% in all in vitro experiments.

Transport inhibition assays using 8-(2-[fluoresceinyl]-aminoethylthio)-adenosine-3′,5′-cyclic-monophosphate (8-FcA) were completed as previously described [23]. OAT1B2 overexpressing cells and vector control cells were grown to confluency in a 96-well plate. Cells were washed, then incubated for 15 min with 100 μL phenol red-free DMEM containing gilteritinib or vehicle (DMSO). The media was removed, then cells were incubated with 45 μL media containing 25 μM 8-FcA for 30 min. The media was removed and cells were washed three times with ice cold PBS. Fluorescence was measured on a plate reader at excitation and emission wavelengths of 485 and 535 nm, respectively. Cells were lysed with 0.2% nitric acid at 4 °C overnight and a Pierce protein assay was used to normalize the data to total protein.

### 4.3. Competitive Counterflow Assays

OATP1B1 overexpressing cells or vector control were seeded in 96-well plates in 200 μL DMEM and incubated for 24 h. The medium was aspirated off and the cells were washed three times with PBS at room temperature. The cells were then treated with 100 μL of 5 μM ACE and incubated at 37 °C for 15 min in uptake buffer. The supernatant was aspirated off and 100 μL of 5 μM ACE alone or in combination with DMSO or gilteritinib (0.1–20 μM) in uptake buffer were added to the cells. The plates were further incubated for 20 min at 37 °C. The supernatant was removed, and the cells were washed three times with 200 μL of ice-cold PBS. Lastly, 200 μL of 0.1 N NaOH was added to cells and incubated for 20 min at room temperature. Fluorescence was measured on a plate reader at excitation and emission wavelengths of 460 and 510 nm, respectively. The intracellular concentrations of ACE were calculated by comparison of fluorescence values observed with control samples incubated with ACE alone, which were set to 100%.

### 4.4. Washout Assay

OATP1B1 overexpressing cells and vector control cells were plated in 12-well plates 24 h prior to experimentation. Cells were then washed with warm PBS and pre-incubated for 15 min with 0.5 mL DMSO or gilteritinib (15 μM) in DMEM at 37 °C. After pre-incubation, the supernatant was removed, and cells were washed with PBS. Compound-free medium was then added, and the cells were further incubated for periods of 15, 30, and 60 min. Next, the medium was removed, and cells were incubated with 0.5 mL of 0.2 μM E*β*G for 15 min. After that, the medium was removed and the cells were washed three times with ice-cold PBS, and 1 N NaOH was added to the wells to stop the rection. The next day 1 M HCL was added to each well and substrate concentrations were assessed using liquid scintillation counting. The resulting data were normalized to total protein as determined by a Pierce protein assay.

### 4.5. In Vivo Pharmacokinetic Studies

Male and female CYP3A(−/−) mice and age- and sex-matched wild-type mice on an FVB/NTac (CYP3A(+/+)) background were bred in-house. Male OATP1B2(−/−) mice and age and sex-matched wild-type mice on a DBA1/lacJ (OATP1B2(+/+)) background were bred in-house [47]. All mice were aged 8–16 weeks and weighed 18–30 g. Littermates of the same sex were housed in groups of five or fewer in a temperature- and light-controlled environment (12-h light/dark cycles) in cages lined with absorbent bedding. Mice were given free access to standard food and water. All breeding and experimental procedures were conducted with the approval of the University Laboratory Animal Resources Animal Care and Use Committee at The Ohio State University under protocol number 201500000101-R2.

For in vivo pharmacokinetic studies, mice were randomly divided into experimental groups of equal size. Gilteritinib powder was dissolved in 0.5% methylcellulose solution in distilled sterile water at a concentration of 2 mg/mL and was administered via oral gavage at a body weight normalized dose of 10 mg/kg. The dose of gilteritinib was chosen based on previous preclinical characterization of gilteritinib pharmacokinetics [31,32]. Itraconazole powder was dissolved in a solvent comprised of 5% DMSO, 70% PEG300, and 25% distilled sterile water and administered orally at a dose of 100 mg/kg. The dose of itraconazole was based on previous work studying preclinical DDI with itraconazole [48]. For combination treatments, itraconazole was always given 30 min prior to gilteritinib. Despite the potential impact of DMSO or PEG300 on gilteritinib pharmacokinetics, the mice that received the vehicle had similar pharmacokinetics to mice that did not receive the vehicle [32].

Pharmacokinetic studies were performed in accordance with a repeat sampling strategy described previously [49]. After a single dose of gilteritinib, 20–40 μL of whole blood samples were collected from each mouse at time points ranging from 0.5–24 h after dosing. Samples procured during the first three time points were collected from the submaxillary vein using a sterile, disposable 4 mm or 5 mm Goldenrod animal blood lancet and collected into a glass micro-hematocrit heparinized capillary tube. For samples obtained at the fourth and fifth time points, the mice were sedated using 2% isoflurane and whole blood was obtained via the retro-orbital venous plexus. At the final time point, mice were euthanized via carbon dioxide inhalation, whole blood was collected by cardiac puncture with a needle and 1-mL syringe and transferred to a 1.5-mL heparinized tube. All samples were centrifuged at 13,000× *g* for 5 min and the plasma supernatant was transferred to a 0.5-mL tube, snap-frozen on dry ice, and stored at −80 °C until the day of analysis.

Pharmacokinetic parameters were estimated using non-compartmental analyses using Phoenix WinNonlin version 8.1 (Certara, Princeton, NJ, USA). Estimated measures of exposure included the peak plasma concentration (C_max_) and the area under the plasma concentration–time curve (AUC).

### 4.6. Statistical Analysis

Experimental data are presented as mean ± SD. Group differences were evaluated for statistical significance using an unpaired Student’s *t*-test (two groups) or two-way ANOVA (>2 groups). Statistical calculations were performed using GraphPad Prism 8.0 (GraphPad Software, San Diego, CA, USA). All statistical tests were two-tailed, and *p* < 0.05 was considered statistically significant.

## Figures and Tables

**Table 1:** Gilteritinib pharmacokinetic parameters.

Mouse Genotype	Co-Treatment Dose (mg/kg)	Gilteritinib C_max_ (ng/mL)	Gilteritinib AUC_0–24h_ (ng ∗ h/mL)	Gilteritinib AUC Fold Increase
Wild-type FVB	None	310 (±180)	4650 (±960)	
Wild-type FVB	Itraconazole (100)	960 (±630) *	11,480 (±4430) **	2.5 (vs. Vehicle)
CYP3A(−/−)	None	380 (±160)	5990 (±1320)	1.3 (vs. WT)
CYP3A(−/−)	Itraconazole (100)	430 (±130)	5840 (±1520)	0.97 (vs. Vehicle)
Wild-type DBA/1J	None	260 (±250)	4550 (±2720)	
OATP1B2(−/−)	None	390 (±380)	6160 (±2250)	1.4 (vs. WT)
OATP1B2(−/−)	None	1430 (±540)	17,840 (±5500)	
OATP1B2(−/−)	Itraconazole (100)	1350 (±1050)	15,050 (±5930)	0.84 (vs. Vehicle)

Each row represents 5–10 male mice dosed with gilteritinib (10 mg/kg; p.o). Values are the mean with SD in parenthesis. Abbreviations: C_max_, maximum plasma concentration; AUC_0–24h,_ area under the concentration–time curve (AUC) from time zero to 24 h; * *p* < 0.05, ** *p* < 0.01.

**Table 2:** CDCA-24G pharmacokinetic parameters.

Mouse Genotype	Treatment (Dose mg/kg)	CDCA-24G C_max_ (ng/mL)	CDCA-24G AUC_0–4h_ (ng ∗ h/mL)	CDCA-24G AUC Fold Increase
Wild-type FVB	None	1.17 (±0.38)	3.78 (±0.20)	
Wild-type FVB	Itraconazole (100)	1.84 (±0.60) *	5.10 (±1.60) *	1.3 (vs. WT) 1.5 (vs. CYP3A (−/−) + Itraconazole)
CYP3A(−/−)	None	1.02 (±0.36)	3.35 (±1.10)	0.9 (vs. WT)
CYP3A(−/−)	Itraconazole (100)	0.98 (±0.20)	3.30 (±1.00)	1 (vs. CYP3A(−/−)) 0.9 (vs. WT Alone)

Each row represents 4–5 female mice. Values are the mean with standard error in parenthesis. Abbreviations: C_max_, maximum plasma concentration; AUC_0–4h,_ area under the concentration–time curve (AUC) from time zero to 4 h (the last observed timepoint); * *p* < 0.05.

## Data Availability

The data presented in this study are available on request from the corresponding author.

## Supplementary Materials

The following supporting information can be downloaded at: https://www.mdpi.com/article/10.3390/molecules27206815/s1, Figure S1: Inhibition of OATP1B1 by itraconazole and metabolites; Figure S2: Expression of CYP3A11 and OATP1B2 in OATP1B2(−/−) and CYP3A(−/−) mice.

## Sample Availability

Samples of the compounds are available from the authors.
