# Supplementary material for: Itraconazole-Induced Increases in Gilteritinib Exposure Are Mediated by CYP3A and OATP1B
Source: Molecules. 2022 Oct 12;27(20):6815. doi: 10.3390/molecules27206815 (PMC9610999; doi:10.3390/molecules27206815)
Supplement: Supplementary file 1 [file molecules-27-06815-s001.zip › molecules-1934207-Supplementary.pdf]

Itraconazole-induced increases in gilteritinib exposure are mediated by CYP3A and OATP1B

Dominique A. Garrison[1], Yan Jin[1], Zahra Talebi[1], Shuiying Hu[1], Alex Sparreboom[1], Sharyn D. Baker[1] and Eric D. Eisenmann[1,*]

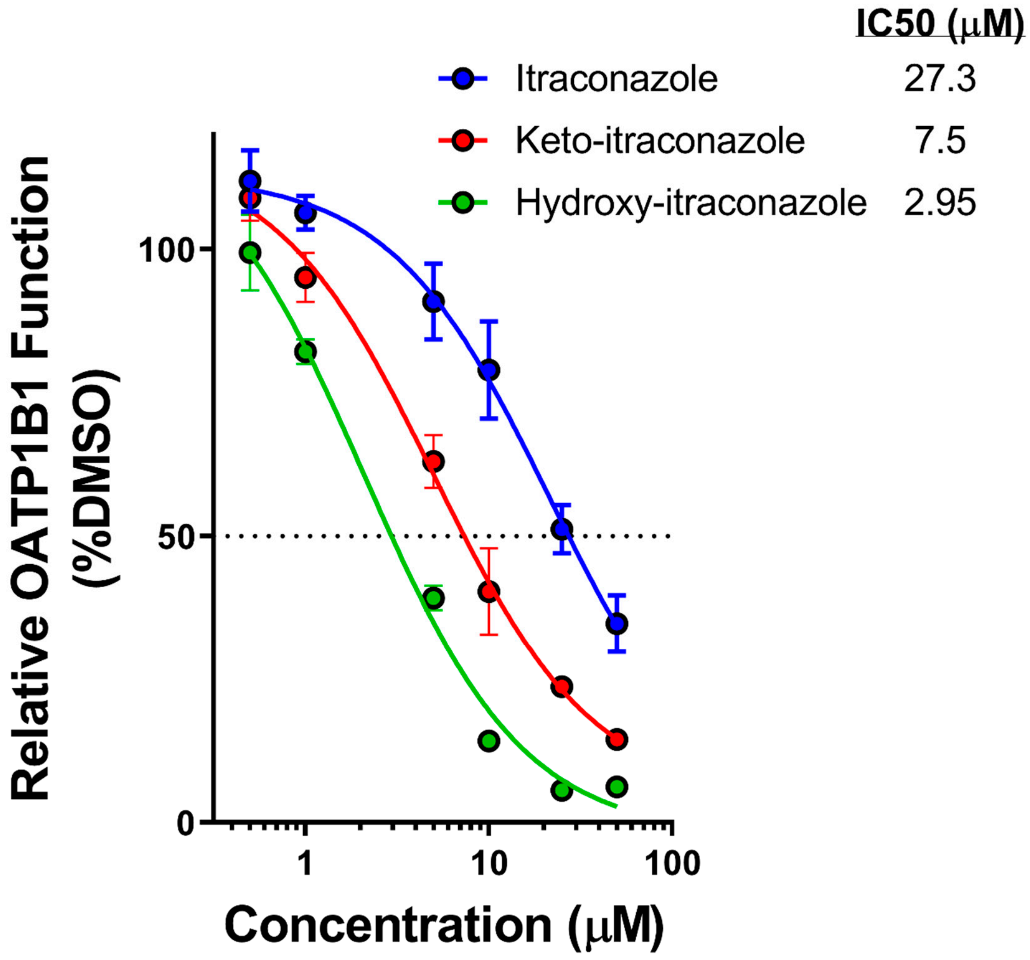

Supplementary Figure S1. Inhibition of OATP1B1 by itraconazole and metabolites. Intracellular accumulation of the radiolabeled probe substrate EβG in the presence of itraconazole, keto-itraconazole, or hydroxy-itraconazole at increasing concentrations (0.5-50 μM).

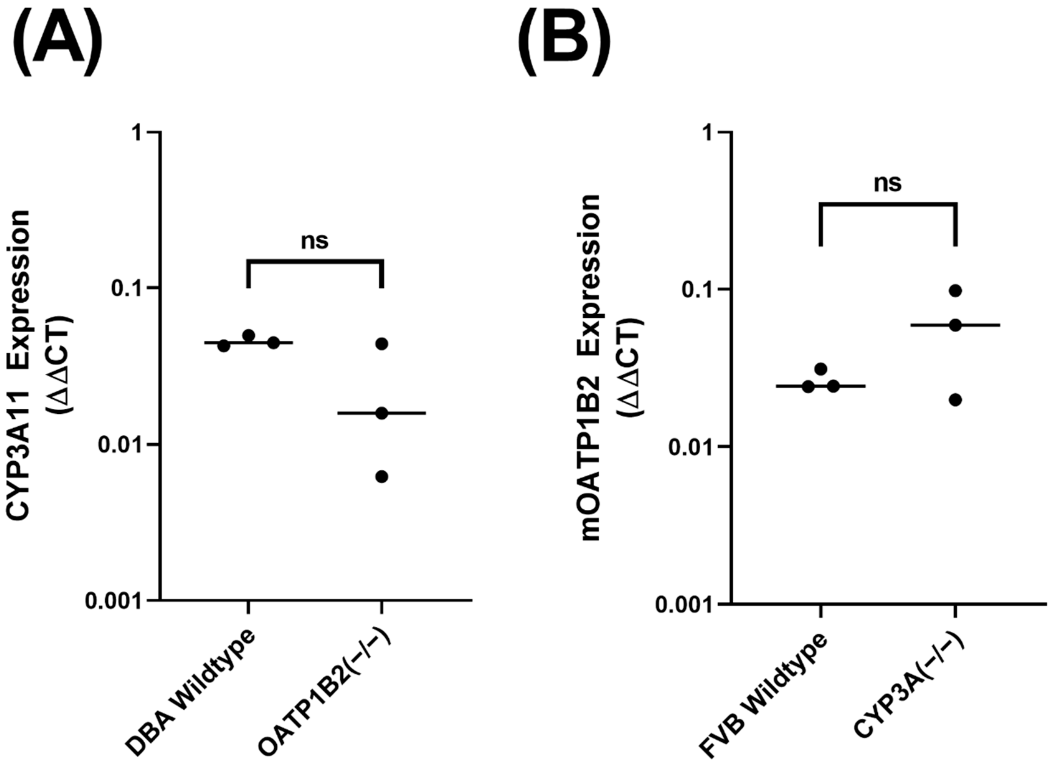

Supplementary Figure S2. Expression of CYP3A11 and OATP1B2 in OATP1B2(−/−) and CYP3A(−/−) mice. Livers were harvested from male CYP3A(−/−), FVB wild-type, OATP1B2(−/−), and DBA wild-type mice between 8 and 12 weeks of age (*n* = 3/group). mRNA expression was assessed by real-time qPCR.
